# The association of dietary insulin load and dietary insulin index with body composition among professional soccer players and referees

**DOI:** 10.1186/s13102-023-00635-1

**Published:** 2023-03-13

**Authors:** Mohammad Beba, Mohammad Gholizadeh, Mohammad Sharifi, Tohid Seifbarghi, Kurosh Djafarian

**Affiliations:** 1 Department of Clinical Nutrition, school of Nutritional Sciences and Dietetics, Tehran University of Medical sciences, P.O. Box 14155-6117, Tehran, Iran; 2 Department of Clinical Nutrition and Dietetics, Faculty of Nutrition and Food Technology, Shahid Beheshti University of Medical Sciences, Tehran, Iran; 3 Department of Sport Medicine, School of Medicine, Tehran University of Medical Sciences, Tehran, Iran

**Keywords:** Dietary insulin index, Dietary insulin load, Body compositions, Soccer players, Soccer referees

## Abstract

**Background:**

There has been limited research undertaken about the association of dietary insulin load (DIL) and dietary insulin index (DII) with body composition in non-athletic adults, however, to the best of our knowledge No previous study has investigated such an association in an athletic population.

**Purpose:**

The aim of this study was to explore the association of DII and DIL with body compositions in male and female soccer players and referees.

**Methods:**

The cross-sectional study was conducted on 199 professional male and female soccer players and referees. A 147-item semi-quantitative food frequency questionnaire (FFQ) was adopted to congregate the participants’ dietary data. Body composition was measured using InBody to gain a detailed understanding of fat mass, percent body fat (PBF), lean mass, percent muscle mass (PMM), and bone mineral content (BMC). Waist circumference (WC), hip circumference (HC), waist-to-hip ratio (WHR), and waist-to-height ratio (WHtR) were obtained from all participants. Other body composition parameters include a body shape index (ABSI), abdominal volume index (AVI), body adiposity index (BAI), body roundness index (BRI), conicity index (CI), weight-adjusted waist index (WWI) and waist-to-hip-to-height ratio (WHHR) were calculated using a particular defined formula.

**Results:**

Results of multiple linear regression revealed that there is a significant association between DIL and BMI (P = 0.04) in < 18 male soccer players, CI (P = 0.04) and WWI (P = 0.03) in ≥ 18 female soccer players, PBF (P = 0.02), PMM (P = 0.01) and WWI (P = 0.01) in ≥ 18 female soccer players. Nevertheless, no significant associations between DIL and body composition parameters were found in the referees. Additionally, there is a significant association between DII and BMC (P = 0.02) in male soccer referees, however, no significant associations were found in young soccer players and female athletes.

**Conclusion:**

This study demonstrates that DIL is positively associated with BMI, CI, and WWI in male soccer players and PBF, and WWI in female soccer players. Although, there was an observed negative association between DIL and PMM in females. In addition, a significant negative association between DII and BMC was observed in male soccer players.

## Introduction

The food insulin index (FII) directly represents the quantity of postprandial insulin secretion after the consumption of a specific food [1]. FII is utilized to demonstrate the ratio of insulin response after a meal to an iso-energetic food’s (such as glucose or white bread) insulin response[1]. Furthermore, dietary insulin load (DIL) and dietary insulin index (DII), are two indices that emblematize insulin response to the total diet [2]. Adherence to unhealthy dietary patterns that induce excessive insulin release lead to beta cell dysfunctions and increase cells’ oxidative stress [3–5]. Diets rich in refined carbohydrates emerged as a strong factor associated with postprandial glucose levels and insulin response [6, 7]. High insulin response accompanies higher fat deposition, elevated lipid profile, and insulin resistance [8, 9]. Insulin resistance stems from the inability of insulin to transport glucose to tissues which may exert an influence on body composition [10, 11]. Insulin resistance proved to be a consequence of disparate non-communicable diseases such as diabetes, obesity, cancers, and cardiovascular diseases [12, 13].

Body composition interprets various elements in the human body [14]. Obesity is commonly evaluated and classified by body mass index (BMI) [15]. Nevertheless, mortality and cardio-metabolic risk factors can differ among individuals with the same BMI [16, 17]. It seems that BMI is not a precise indicator to assess the odds of mortality and non-communicable disease risk factors. Furthermore, fat distribution and muscle mass are more accurate indicators for health and morbidity than BMI alone [18, 19]. The most prominent fat depot is visceral adipose tissue, which is implicated in unbalanced lipid profile, fasting blood glucose, and metabolic syndrome, but be that as it may, subcutaneous adipose tissue can also be protective [20, 21]. Moreover, increased body fat and sedentary lifestyles cause mitochondrial dysfunction and insulin resistance[22, 23].

Exercise reduces the process of sarcopenia (decreasing muscle mass and increasing body fat) by protecting muscle mass and reducing fat deposition. This mechanism diminishes many inflammatory factors and insulin resistance [24, 25].

Exercise improves protein synthesis rate and helps to maintain fat-free mass [26, 27]. Besides, it increases muscle function, enhances insulin responsiveness, leads to GLUT-4 expression, and promotes oxidative capacity. In addition, exercise is demonstrated the greatest impacts on oxidative fiber and several cytokines, adipokine such as leptin, as well as increasing fatty acids oxidation and decreasing muscle fat depositions. [28, 29]. Soccer is more enjoyable and sought-after than other kinds of training. Consistent exercise upholds muscle insulin sensitivity and increases adaptive response by promoting muscle size, capillarization, morphology, and protein composition. Whereas, adaptation protects insulin sensitivity and has a health-promoting effect. Animal studies have presented that exercise elevates insulin-stimulated glucose uptake via the AMPK-dependent form. [28, 30]. Some studies have shown an increasing whole-body insulin sensitivity in exercise [30, 31]. Up to now, far too little attention has been paid to the association of DIL and DII with body composition.

Some previous studies performed in this area found a significant correlation between dietary insulin index and dietary insulin loud with insulin resistance[32]. It has previously been observed that postprandial insulin showed an unfavorable effect on body composition in young adulthood [33]. More exercise increases insulin-sensitizing and protects fat-free body mass by activation of AMPK [30]. So far, however, the relevance and association of body composition with dietary insulin index and dietary insulin have remained unclear. Therefore, The aim of this study is to explore the relationship between dietary insulin load and dietary insulin index with body compositions indices (BMI, fat percent, fat-free mass (FFM), fat mass, percent body fat, lean mass, percent muscle mass, bone mineral content, Waist-to-hip ratio (WHR), Waist-to-height ratio (WHtR), waist circumference, hip circumference (cm), a body shape index (ABSI), Abdominal volume index (AVI), Body adiposity index (BAI), Body roundness index (BRI), Weight-adjusted waist index (WWI) and Waist-to-hip-to-height ratio (WHHR)) among professional soccer players and referees.

## Materials and methods

### Study population and design

The cross-sectional study was carried out among 199 elites (11 males and 22 females) and sub-elite (13 males and 24 females) soccer players and elite referees (90 males and 39 females) in Iran, during the early stages of the 2019–2020 competitive season. Elite and sub-elite (the national under-18 soccer players) soccer players as well as elite soccer referees from all divisions, under the directive of The Football Federation Islamic Republic of Iran, were recruited for this study. Data including participants’ demographics (age, gender, and education), physical activity, medical history, anthropometric measurements, and dietary intake were gathered via a face-to-face interview with mentioned athletes. Informed consent was obtained from all the participants and their legal guardians. This study was conducted according to the guidelines laid down in the Declaration of Helsinki and all procedures involving human subjects were approved by the Ethics Committee of Tehran University of Medical Sciences; (Ethic number: IR.TUMS.VCR.REC.1398.729)]. Subjects were all given verbal and written communication about the study before signing an informed consent form.

Convenience sampling was used for this study, and all players and referees who agreed to participate in the present study were included in the study. We used Brooke L Devlin’s study to calculate the required sample size [34]. Fat-free mass (FFM) was reckoned to be the largest sample size among other variables. Therefore, we calculated the study power based on this variable. Thus, with a power of 80%, type I error of 0.05, desired confidence interval (CI) of 0.95, and effect size (d) of 0.86, the minimum required sample size was estimated to be 11 subjects, but since we were working on different categories of athletes (under/over 18 years, male/female, soccer players/referees) and on account of over- or under-reporting by some individuals and ruling out the possibility of missing some information, the eventual sample size consisted of 199 subjects. The main reason both soccer players and referees were analyzed together was that, while the soccer players play the most influential role in this popular sport[35, 36], the soccer referees also play a critical role in the modern era[37–40]. Furthermore, the physical activity level of soccer referees during a match has been estimated to be around 10-12 km, with 4–18% of this match distance covered at speeds faster than 13-15 km/h[41], which is almost equal to what is observed in midfield players[35–42]. Collection of all information related to anthropometric indices, demographic and lifestyle factors include dietary intake and physical activity took place at the Medical Committee of the Football Federation Islamic Republic of Iran.

### Assessment of Dietary intake

A 147-item semi-quantitative food frequency questionnaire (FFQ) validated in the Tehran Lipid and Glucose Study (TLGS) was adopted to congregate the participants’ dietary data. The validity and reliability of this 147-item semi-quantitative questionnaire have been published elsewhere [43]. This questionnaire elicits food intakes of the past year; subsequently, dietary intake data was then entered into Nutritionist IV software modified for Iranian foods to estimate nutrient intake composition. Average energy, macro and micro-nutrient intakes were also acquired.

### Dietary insulin load (DIL) and dietary insulin index (DII) calculation

Food insulin index (FII) is the area under the curve that causes insulin surge within 2 h after consuming 1000 kj (239 kcal) of a specific food divided by the area under the curve after consuming 1000 kj of a reference food (e.g. white bread). The FII for each food item was procured from previous studies by Holt et al., Bao et al., and Bell et al. [1, 2, 44]. Since the FII of all Iranian dishes was not available in the food list of mentioned studies, the FII of similar food items was used. We used the following formula to calculate the insulin load of each food:

Insulin load of a given food = FII of that food × the energy content of that food per 1 gram (kcal) × an amount of that food consumed in a day (gr/day), which is proposed by Nimptsch et al. [45]. Total dietary insulin load for each participant was computed by summing the insulin load of all foods consumed by that participant. Finally, the DII for each participant was calculated by dividing the DIL by the total energy consumed by that person.

### Assessment of body composition

Body composition was measured using the InBody 570 (InBody Co., Ltd. in Seoul, Korea), and analyzed to quantify fat mass, percent body fat, lean mass, percent muscle mass, and bone mineral content. The InBody 570 uses three different frequencies (5 kHz, 50 kHz, 500 kHz) at each of five segments (right arm, left arm, trunk, right leg, and left leg). Calibration took place as per manufacturer guidelines. Participants’ measurements were taken after an overnight fast and rest, without exercise, on the morning of the scan. Participants were required to empty their bladders and be minimally clothed before each scan. Athletes were advised not to consume caffeinated beverages at least 4 h before and drink at least 2–4 glasses of water 2 h before scanning. The software automatically analyzed scans. Body weight was measured with subjects in light clothing, upshot, using a digital scale (Seca 808, Germany) to the nearest 0.1 kg, whilst height was assessed using a wall-mounted stadiometer (Seca, Germany) to the nearest 0.1 cm. BMI was calculated by dividing weight (kg) by the square of height(m). Waist circumference (WC) was measured at the midpoint of the lowest rib and iliac crest at the end of expiration using a non-elastic measuring tape (Seca 201, Germany) to the nearest 0.1 cm. Hip circumference (HC) was measured at the widest point over the buttocks using a measuring tape to the nearest 0.1 cm. Waist-to-hip ratio (WHR) and Waist-to-height ratio (WHtR) were obtained by dividing the waist circumference (cm) by the hip circumference (cm) and height (cm), respectively. Other body composition parameters include A body shape index (ABSI), Abdominal volume index (AVI), Body adiposity index (BAI), Body roundness index (BRI), Conicity index (CI), Weight-adjusted waist index (WWI) and Waist-to-hip-to-height ratio (WHHR) were calculated using a specific formula published by Chang et al[46].

### Assessment of physical activity

A 7-item (short form) International Physical Activity Questionnaire (IPAQ) was employed to ascertain the participants’ physical activity levels. The validity and reliability of this questionnaire have been described and confirmed elsewhere [47]. This questionnaire asks the participants about the types of physical activities performed in the preceding 7 days. Individuals were divided into 3 groups in terms of physical activity:

1. Low activity: This group does not meet any of the criteria for subsequent groups.
2. Average: Having any type of physical activity (light, moderate or heavy) for 5 days or more in a week to meet 600MET/minute/week.
3. High activity: Having any type of physical activity (light, moderate or heavy) for 7 days a week to meet 3000MET/minute/week.

### Statistical methods

The R Studio software (Version 2022.07.1) [48] was used for all statistical analyses and statistical significance was set at p **<** 0.05. Descriptive statistics (Frequencies, cross-tabulation, and Chi-square value) were used to elucidate the primary features of the data. Participants’ general characteristics were compared across tertiles of Dietary Insulin Load (DIL) and Dietary Insulin Index (DII) using an analysis of variance (ANOVA) for continuous variables. Pearson’s correlation coefficient was used to discern the correlation between DIL and DII with the measures of body composition. To identify associations between DIL and DII with body composition parameters (Body Mass Index (BMI), Percent Body Fat (PBF), Percent Muscle Mass (PMM), Waist to Hip Ratio (WHR), Waist to Height Ratio (WHtR), Bone Mineral Content (BMC), A body shape index (ABSI), Abdominal volume index (AVI), Body adiposity index (BAI), Body roundness index (BRI), Conicity index (CI), Weight-adjusted waist index (WWI) and Waist-to-hip-to-height ratio (WHHR)), multivariate regression models were created, with adjustment for potential covariates such as age, gender, and physical activity. The power of 80%, type I error of 0.05, desired confidence interval (CI) of 0.95, and effect size (d) of 0.86 was used for the statistical analyses.

All variables were tested for normality via the Kolmogorov-Smirnov statistic and visual assessment of histograms, and appropriate statistical tests were subsequently conducted. Data are presented as percentages, means, and standard deviations.

## Results

The general characteristics of soccer players and referees are indicated in Table 1. However, to better display the results, it was decided to show the characteristics of participants across the tertiles of DIL and DII are demonstrated in Table 2A and Table 2B, respectively. All 199 volunteers partook in the present study, consisting of 113 (56.8%) males and 86 (43.2%) females. The mean age of participants was 29.38 ± 8.53 years, of which 36 (18.1%) were under 18 and 163 (81.9%) were over 18 years of age. Of all the participants, 70 (35.2%) were soccer players and 129 (64.8%) were soccer referees. Mean physical activity was 3003.75 ± 1834.97 MET/min/week, and, according to this, 144 (72.4%) obeyed a moderate physical activity, and 55 (27.6%) followed a high physical activity lifestyle. A significant difference in mean DIL, age, post position, CI, total calorie intake, carbohydrate intake, protein intake, and fat intake is apparent from the tertiles of DIL. Results of Tukey’s test quite revealed a significant difference between tertile 1, tertile 2, and tertile 3 in the case of mean DIL, while, regarding age and post position, there is a significant difference among tertiles. CI was also significantly different in tertiles 1 and 3. Total calorie, carbohydrate, protein, and fat intake were also significantly different among all tertiles of DIL. No significant differences in the mean and frequency of other characteristics were evident (P > 0.005). Additionally, results of Post Hoc analysis on DII illustrated that there is a significant difference between mean DII, physical activity, BMI, WC, AVI, and fat intake. Mean DII was significantly different across all tertiles of DII. Further, physical activity, BMI, WC, and AVI were significantly different between tertile 1 and tertile 2 of DII. Furthermore, total fat intake was also different among tertile 1 and tertile 3 of DII, according to Tukey’s test. There were no significant differences in the mean and frequency of other characteristics (P > 0.005).

**Table 1:** General characteristics of soccer players and referees

Variables	Soccer players				Soccer referees			
	**Male** **(N = 23)**		**Female** **(N = 47)**		**Male** **(N = 90)**		**Female** **(N = 39)**	
	Mean	SD	Mean	SD	Mean	SD	Mean	SD
**Mean DIL** ^a^	209542.49	63824.56	155340.01	72999.52	136276.27	53411.98	159335.35	126806.33
**Mean DII** ^a^	53.20	4.84	51.47	7.66	52.76	7.69	49.87	7.06
**Age (year)** ^a^ *< 18 years* *≥ 18 years*	18.29 12 (52%) 11 (48%)	3.36	19.93 24 (51%) 23 (49%)	4.61	35.47 90 (100%)	4.14	33.28 39 (100%)	4.23
**Physical activity** ^b^ *Moderate* *High*	20 (87%) 3 (13%)		40 (85%) 7 (15%)		52 (58%) 38 (42%)		32 (82%) 7 (18%)	
**Physical activity** ^a^ MET/Min/Week	2348.29	1195.55	2730.19	2098.07	3242.86	1527.90	3178	2338.89
**Height (cm)** ^a^	176.41	5.89	167.17	6.69	177.92	6.05	164.49	5.44
**Weight (kg)** ^a^	68.17	7.63	59.43	6.58	74.56	6.61	59.45	6.07
**BMI (kg/m**^2^)^a^	21.82	1.64	21.22	1.88	23.48	1.60	22.03	2.28
**BFM (kg)** ^a^	9.16	3.23	12.26	3.26	12.77	3.20	15.17	4.09
**PBF (%) ** ^a^	13.43	4.40	20.60	4.81	17.04	3.75	25.29	5.14
**FFM (Kg)** ^a^	59.00	7.06	47.14	5.95	61.79	5.53	44.27	4.22
**PMM (%)** ^a^	86.57	4.41	79.40	4.81	82.95	3.74	74.69	5.14
**WC** ^a^	75.16	4.49	74.84	4.69	82.06	4.62	76.17	5.33
**HC** ^a^	95.43	3.54	92.39	3.58	98.33	3.17	93.26	3.83
**WHR** ^a^	0.78	0.03	0.81	0.04	0.83	0.03	0.81	0.03
**WHtR** ^a^	0.42	0.02	0.44	0.03	0.46	0.02	0.46	0.03
**ABSI** ^a^	0.07	0.003	0.07	0.003	0.07	0.002	0.07	0.002
**AVI** ^a^	11.63	1.30	11.47	1.35	13.70	1.45	11.87	1.63
**BAI** ^a^	22.76	1.71	24.85	2.84	23.49	2.07	26.29	2.82
**BRI** ^a^	2.09	0.41	2.46	0.53	2.68	0.46	2.72	0.64
**CI** ^a^	1.11	0.04	1.15	0.04	1.16	0.04	1.16	0.04
**WWI ** ^a^	9.12	0.43	9.72	0.41	9.51	0.37	9.88	0.39
**WHHR** ^a^	0.44	0.02	0.48	0.03	0.46	0.02	0.49	0.02
**BMC (kg) ** ^a^	3.43	0.44	2.82	0.40	3.50	0.38	2.64	0.28
**Calorie intake (Kcal) ** ^a^	3952.57	1208.27	3055.98	1532.86	2634.83	1107.82	3195.89	2457.89
**Carbohydrates (gr/d)** ^a^	628.66	204.20	458.53	252.45	404.42	177.40	516.53	622.96
**Protein intake (g/d) ** ^a^	146.20	36.44	121.40	64.08	108.70	53.97	115.45	41.26
**Fat intake (g/d)** ^a^	112.19	41.98	92.56	47.53	75.65	33.74	92.92	41.76

ABSI = A body shape index, AVI = Abdominal volume index, BAI = Body adiposity index, BFM = Body fat mass, BMC = Bone mineral content, BMI = Body mass index, BRI = Body roundness index, CI = Conicity index, FFM = Fat free mass, HC = Hip circumference, PBF = Percent body fat, PMM = Percent muscle mass, WC = Waist circumference, WHR = Waist to hip ratio, WHHR = Waist to hip to height ratio, WHtR = Waist to height ratio, WWI = Weight-adjusted-waist-index

^a^ Values are expressed as mean ± SD, by using one-way ANOVA

^b^ Values are reported as total and percentage, by using cross-tabulation and Chi-square test

**Table 2A:** General characteristics of participants across tertiles of Dietary Insulin Load (DIL)

	Dietary Insulin Load								P-value
	Tertile1 (N = 66)		Tertile2 (N = 67)		Tertile3 (N = 66)		Total (N = 199)		
	Mean	SD	Mean	SD	Mean	SD	Mean	SD	
**Mean DIL** ^a^	92950.21	15733.17	140767.95	15677.28	228597.53	99586.04	154038.21	81,089	**< 0.001**
**Age (year)** ^a^ *< 18 years* *≥ 18 years*	31.65 8 (12.1%) 58 (87.9%)	7.5	30 8 (11.9%) 59 (88.1%)	8.68	26.47 20 (30.3%) 46 (69.7%)	8.64	29.38 36 (18.1%) 163 (81.9%)	8.53	**0.002**
**Gender** ^b^ *Male* *Female*	38 (57.6%) 28 (42.4%)		36 (53.7%) 31 (46.3%)		39 (59.1%) 27 (40.9%)		113 (56.8%) 86 (43.2%)		0.81
**Post** ^b^ *Soccer players* *Soccer referees*	15 (22.7%) 51 (77.3%)		22 (32.8%) 45 (67.2%)		32 (49.2%) 33 (50.8%)		70 (35.2%) 129 (64.8%)		**< 0.001**
**Physical activity** ^b^ *Moderate* *High*	45 (68.2%) 21 (31.8%)		49 (73.1%) 18 (26.9%)		50 (75.8%) 16 (24.2%)		144 (72.4%) 55 (27.6%)		0.61
**Physical activity** ^a^ MET/Min/Week	3133.73	1750.47	2873.88	1720.29	3005.62	2.37.92	3003.75	1834.97	0.71
**Height (cm)** ^a^	172.63	9.45	172.36	7.79	172.87	8.03	172.62	8.41	0.94
**Weight** ** (kg)** ^a^	67.63	9.93	67.37	9.95	66.99	9.31	67.33	9.69	0.93
**BMI (kg/m**^2^) ^a^	22.64	2.12	22.58	1.88	22.20	2.13	22.47	2.04	0.41
**BFM (kg)** ^a^	13.03	3.69	12.97	2.97	12.05	4.49	12.69	3.77	0.25
**PBF (%)** ^a^	19.55	5.93	19.46	4.37	18.12	6.63	19.05	5.73	0.27
**FFM (Kg)** ^a^	54.6	10.12	54.39	9.36	54.93	9.20	54.64	9.52	0.055
**PMM (%) ** ^a^	80.44	5.93	80.54	4.37	81.88	6.64	80.95	5.73	0.27
**WC** ^a^	79.03	5.49	78.84	5.95	77.33	5.88	78.40	5.80	0.18
**HC** ^a^	95.77	4.22	95.76	4.17	95.31	4.58	95.62	4.31	0.77
**WHR** ^a^	0.82	0.03	0.82	0.03	0.81	0.04	0.81	0.03	0.09
**WHtR** ^a^	0.45	0.03	0.45	0.02	0.44	0.03	0.45	0.03	0.11
**ABSI** ^a^	0.075	0.002	0.075	0.003	0.074	0.003	0.075	0.003	0.34
**AVI** ^a^	12.75	1.67	12.71	1.84	12.26	1.78	12.57	1.77	0.21
**BAI** ^a^	24.38	3.03	24.40	2.25	24.03	2.65	24.27	2.66	0.66
**BRI** ^a^	2.63	0.53	2.61	0.49	2.46	0.61	2.57	0.55	0.14
**CI** ^a^	1.16	0.03	1.16	0.04	1.14	0.05	1.15	0.04	**0.03**
**WWI** ^a^	9.64	0.39	9.63	0.37	9.48	0.54	9.58	0.45	0.059
**WHHR** ^a^	0.478	0.028	0.478	0.024	0.047	0.036	0.475	0.03	0.22
**BMC (kg)** ^a^	3.15	0.55	3.16	0.50	3.20	0.54	3.17	0.53	0.85
**Calorie intake (Kcal)** ^a^	1881.36	399.79	2735.88	479.56	4389.96	2050.08	3001.06	1610.45	**< 0.001**
**Carbohydrates (gr/d)** ^a^	277.20	65.81	413.97	81.23	707.44	486.04	465.94	336.75	**< 0.001**
**Protein intake (g/d)** ^a^	76.48	21.08	112.67	25.04	163.35	61.42	117.48	53.50	**< 0.001**
**Fat intake (g/d)** ^a^	58.36	18.28	81.65	23.44	122.13	47.05	87.35	41.40	**< 0.001**

ABSI = A body shape index, AVI = Abdominal volume index, BAI = Body adiposity index, BFM = Body fat mass, BMC = Bone mineral content, BMI = Body mass index, BRI = Body roundness index, CI = Conicity index, FFM = Fat free mass, HC = Hip circumference, PBF = Percent body fat, PMM = Percent muscle mass, WC = Waist circumference, WHR = Waist to hip ratio, WHHR = Waist to hip to height ratio, WHtR = Waist to height ratio, WWI = Weight-adjusted-waist-index

^a^ Values are expressed as mean ± SD, by using one-way ANOVA

^b^ Values are reported as total and percentage, by using cross-tabulation and Chi-square test

P-value is considered significant at < 0.05

**Table 2B:** General characteristics of participants across tertiles of Dietary Insulin Index (DII)

	Dietary Insulin Index								P-value
	Tertile1 (N = 66)		Tertile2 (N = 67)		Tertile3 (N = 66)		Total (N = 199)		
	Mean	SD	Mean	SD	Mean	SD	Mean	SD	
**Mean DII** ^a^	44.43	3.67	51.31	1.67	60.10	4.69	51.94	7.32	**< 0.001**
**Age (year)** ^a^ *< 18 years* *≥ 18 years*	28.77 13 (19.7%) 53 (80.3%)	8.93	30.37 10 (14.9%) 57 (85.1%)	7.89	28.97 13 (19.7%) 53 (80.3%)	8.77	29.38 36 (18.1%) 163 (81.9%)	8.53	0.50
**Gender** ^b^ *Male* *Female*	32 (48.5%) 34 (51.5%)		42 (62.7%) 25 (37.3%)		39 (59.1%) 27 (40.9%)		113 (56.8%) 86 (43.2%)		0.23
**Post** ^b^ *Soccer players* *Soccer referees*	26 (39.3%) 40 (60.7%)		17 (25.37%) 50 (74.63%)		27 (40.9%) 39 (39.1%)		70 (35.2%) 129 (64.8%)		0.32
**Physical activity** ^b^ *Moderate* *High*	51 (77.3%) 15 (22.7%)		44 (65.7%) 23 (34.3%)		49 (74.2%) 17 (25.8%)		144 (72.4%) 55 (27.6%)		0.30
**Physical activity** ^a^ MET/Min/Week	2679.19	1411.14	3510.78	2325.52	2813.61	1538.02	3003.75	1834.97	**0.01**
**Height (cm)** ^a^	171.71	8.44	172.88	8.67	173.27	8.16	172.62	8.41	0.54
**Weight (kg)** ^a^	65.16	9.43	68.58	9.39	68.23	10.02	67.33	9.69	0.08
**BMI (kg/m**^2^)^a^	21.93	2.07	22.81	1.97	22.67	2.01	22.47	2.04	**0.02**
**BFM (kg)** ^a^	12.03	3.30	13.03	4.39	12.99	3.50	12.69	3.77	0.22
**PBF (%)** ^a^	18.70	5.24	19.12	6.35	19.32	5.59	19.05	5.72	0.81
**FFM (Kg) ** ^a^	53.13	9.36	55.55	9.14	55.23	10.01	54.64	9.52	0.28
**PMM (%)** ^a^	81.30	5.23	80.87	6.36	80.67	5.60	80.95	5.73	0.81
**WC** ^a^	76.82	5.33	79.29	6.05	79.10	5.75	78.40	5.80	**0.02**
**HC** ^a^	94.63	4.19	96.26	4.14	95.94	4.48	95.62	4.31	0.06
**WHR** ^a^	0.81	0.034	0.82	0.036	0.82	0.041	0.81	0.038	0.10
**WHtR ** ^a^	0.447	0.030	0.459	0.033	0.456	0.030	0.454	0.032	0.10
**ABSI** ^a^	0.075	0.003	0.075	0.002	0.075	0.003	0.075	0.003	0.95
**AVI** ^a^	12.08	1.60	12.85	1.84	12.79	1.77	12.57	1.77	**0.02**
**BAI** ^a^	24.18	2.79	24.47	2.78	24.15	2.40	24.27	2.66	0.74
**BRI** ^a^	2.45	0.52	2.64	0.58	2.60	0.53	2.57	0.55	0.10
**CI** ^a^	1.14	0.046	1.15	0.046	1.16	0.049	1.15	0.047	0.27
**WWI** ^a^	9.55	0.43	9.60	0.45	9.61	0.46	9.58	0.45	0.71
**WHHR** ^a^	0.473	0.029	0.476	0.029	0.476	0.032	0.475	0.030	0.78
**BMC (kg)** ^a^	3.08	0.50	3.21	0.51	3.21	0.57	3.17	0.53	0.25
**Calorie intake (Kcal)** ^a^	3177.35	1479.90	3176	2109.80	2647.18	1000.15	3001.06	1610.45	0.09
**Carbohydrates (gr/d)** ^a^	470.18	242.67	513.87	498.37	413.05	170.66	465.94	336.75	0.22
**Protein intake (g/d)** ^a^	126.63	58.86	120.34	59.74	105.42	37.28	117.48	53.50	0.06
**Fat intake (g/d)** ^a^	102.03	48.09	86.65	38.81	73.38	31	87.35	41.40	**< 0.001**

ABSI = A body shape index, AVI = Abdominal volume index, BAI = Body adiposity index, BFM = Body fat mass, BMC = Bone mineral content, BMI = Body mass index, BRI = Body roundness index, CI = Conicity index, FFM = Fat free mass, HC = Hip circumference, PBF = Percent body fat, PMM = Percent muscle mass, WC = Waist circumference, WHR = Waist to hip ratio, WHHR = Waist to hip to height ratio, WHtR = Waist to height ratio, WWI = Weight-adjusted-waist-index

^a^ Values are expressed as mean ± SD, by using one-way ANOVA

^b^ Values are reported as total and percentage, by using cross-tabulation and Chi-square test

P-value is considered significant at < 0.05

The correlations between DIL/DII and body composition parameters are presented in the order in Table 3A and Table 3B. Our crude model showed significant correlations between DIL and PBF (r = 0.60, P = 0.04), PMM (r = -0.61, P = 0.04), WHR (r = 0.67, P = 0.02), ABSI (r = 0.67, P = 0.02), CI (r = 0.72, P = 0.01) and WWI (r = 0.66, P = 0.02) in ≥ 18 male soccer players, and PBF (r = -0.49, P = 0.02), PMM (r = 0.49, P = 0.02), WHR (r = -0.43, P = 0.04) and WWI (r = -0.53, P = 0.01) in ≥ 18 female soccer players. Conversely, we did not find any significant correlation between DIL and body composition parameters among other athletes. A significant correlation was likewise obvious between DII and BAI (r = 0.64, P = 0.01) in < 18 male soccer players, CI (r = 0.41, P = 0.04) in < 18 female soccer players, and BMC (r = 0.24, P = 0.01) in male soccer referees. On the other hand, no evidence of a considerable correlation between DII and body composition parameters among other athletes was detected.

**Table 3A:** Correlation between Dietary Insulin Load (DIL) and measures of body composition among different categories of athletes

Categories of Athletes	BMI r^a^(*P*)^b^	PBF r^a^(*P*)^b^	PMM r^a^(*P*)^b^	WC r^a^(*P*)^b^	HC r^a^(*P*)^b^	WHR r^a^(*P*)^b^	WHtR r^a^(*P*)^b^	ABSI r^a^(*P*)^b^	AVI r^a^(*P*)^b^	BAI r^a^(*P*)^b^	BRI r^a^(*P*)^b^	CI r^a^(*P*)^b^	WWI r^a^(*P*)^b^	WHHR r^a^(*P*)^b^	BMC r^a^(*P*)^b^
**< 18 male soccer players**	-0.34 (0.24)	-0.39 (0.17)	0.39 (0.17)	-0.40 (0.16)	-0.37 (0.20)	-0.25 (0.41)	-0.34 (0.25)	*0.12* (*0.68*)	-0.41 (0.16)	-0.17 (0.56)	-0.34 (0.25)	-0.22 (0.46)	-0.16 (0.59)	-0.12 (0.68)	-0.35 (0.23)
**≥ 18 male soccer players**	0.07 (0.82)	**0.60** **(0.04** ^*^ **)**	**-0.61** **(0.04** ^*^ **)**	0.45 (0.16)	-0.01 (0.97)	**0.67** **(0.02** ^*^ **)**	0.54 (0.08)	**0.67** **(0.02** ^*^ **)**	0.42 (0.19)	0.17 (0.61)	0.54 (0.08)	**0.72** **(0.01** ^*^ **)**	**0.66** **(0.02** ^*^ **)**	0.57 (0.06)	-0.18 (0.58)
**< 18 female soccer players**	-0.18 (0.38)	0.02 (0.92)	-0.01 (0.93)	-0.13 (0.51)	-0.17 (0.41)	-0.02 (0.92)	-0.11 (0.59)	0.06 (0.77)	-0.13 (0.53)	-0.10 (0.61)	-0.10 (0.61)	-0.02 (0.92)	-0.00 (0.99)	0.00 (0.98)	-0.09 (0.66)
**≥ 18 female soccer players**	-0.26 (0.24)	**-0.49** **(0.02** ^*^ **)**	**0.49** **(0.02** ^*^ **)**	-0.32 (0.14)	-0.09 (0.68)	**-0.43** **(0.04** ^*^ **)**	-0.32 (0.13)	-0.17 (0.43)	-0.31 (0.16)	-0.15 (0.50)	-0.32 (0.14)	-0.36 (0.09)	**-0.53** **(0.01** ^*^ **)**	-0.38 (0.07)	0.40 (0.06)
**Male soccer referees**	0.01 (0.88)	-0.08 (0.45)	0.08 (0.44)	-0.11 (0.29)	-0.03 (0.75)	-0.13 (0.21)	-0.4 (0.66)	-0.14 (0.16)	-0.10 (0.31)	0.08 (0.41)	-0.04 (0.66)	-0.13 (0.19)	-0.08 (0.44)	-0.03 (0.77)	-0.06 (0.56)
**Female soccer referees**	-0.07 0.65	-0.05 0.72	0.05 0.72	0.04 0.80	-0.05 0.72	0.13 0.43	0.01 0.91	0.20 0.20	0.03 0.84	-0.07 0.65	0.01 0.94	0.11 0.48	0.08 0.60	0.07 0.66	0.00 0.99

ABSI = A body shape index, AVI = Abdominal volume index, BAI = Body adiposity index, BFM = Body fat mass, BMC = Bone mineral content, BMI = Body mass index, BRI = Body roundness index, CI = Conicity index, FFM = Fat free mass, HC = Hip circumference, PBF = Percent body fat, PMM = Percent muscle mass, WC = Waist circumference, WHR = Waist to hip ratio, WHHR = Waist to hip to height ratio, WHtR = Waist to height ratio, WWI = Weight-adjusted-waist-index

^a^ ‘r’: Pearson correlation coefficient

^b^ P: significant value

Effect size = 0.86, confidence interval = 0.95

*. Correlation is significant at the 0.05 level (2-tailed)

**Table 3B:** Correlation between Dietary Insulin Index (DII) and measures of body composition among different categories of athletes

Categories of Athletes	BMI r^a^(*P*)^b^	PBF r^a^(*P*)^b^	PMM r^a^(*P*)^b^	WC r^a^(*P*)^b^	HC r^a^(*P*)^b^	WHR r^a^(*P*)^b^	WHtR r^a^(*P*)^b^	ABSI r^a^(*P*)^b^	AVI r^a^(*P*)^b^	BAI r^a^(*P*)^b^	BRI r^a^(*P*)^b^	CI r^a^(*P*)^b^	WWI r^a^(*P*)^b^	WHHR r^a^(*P*)^b^	BMC r^a^(*P*)^b^
**< 18 male soccer players**	0.46 (0.11)	0.05 (0.86)	-0.05 (0.85)	0.12 (0.67)	0.28 (0.34)	-0.08 (0.78)	0.30 (0.31)	-0.21 (0.48)	0.15 (0.62)	**0.64** **(0.01** ^*^ **)**	0.30 (0.31)	-0.09 (0.75)	0.02 (0.94)	0.13 (0.67)	0.00 (0.99)
**≥ 18 male soccer players**	-0.29 (0.37)	-0.14 (0.66)	0.14 (0.66)	-0.22 (0.50)	-0.00 (0.99)	-0.32 (0.33)	-0.48 (0.12)	-0.13 (0.69)	-0.23 (0.49)	-0.55 (0.07)	-0.49 (0.12)	-0.20 (0.54)	-0.36 (0.27)	-0.50 (0.11)	0.18 (0.58)
**< 18 female soccer players**	-0.05 (0.81)	0.21 (0.30)	-0.22 (0.30)	0.29 (0.17)	-0.06 (0.76)	0.38 (0.06)	0.17 (0.41)	0.38 (0.06)	0.27 (0.19)	-0.26 (0.21)	0.17 (0.41)	**0.41** **(0.04** ^*^ **)**	0.28 (0.18)	0.21 (0.31)	-0.01 (0.95)
**≥ 18 female soccer players**	0.30 (0.16)	0.30 (0.16)	-0.30 (0.16)	0.12 (0.57)	0.09 (0.67)	0.13 (0.55)	0.08 (0.71)	-0.20 (0.36)	0.12 (0.57)	0.01 (0.94)	0.08 (0.72)	0.27 (0.22)	0.31 (0.15)	0.06 (0.76)	-0.20 (0.36)
**Male soccer referees**	0.12 (0.22)	-0.06 (0.56)	0.06 (0.57)	0.16 (0.11)	0.17 (0.10)	0.09 (0.40)	0.03 (0.75)	-0.01 (0.90)	0.17 (0.10)	-0.11 (0.30)	0.03 (0.75)	0.07 (0.49)	-0.02 (0.81)	-0.07 (0.48)	**0.24** **(0.01** ^*^ **)**
**Female soccer referees**	-0.02 (0.86)	0.04 (0.79)	-0.04 (0.78)	0.13 (0.43)	0.03 (0.84)	0.18 (0.26)	0.05 (0.73)	0.23 (0.14)	0.13 (0.43)	-0.09 (0.56)	0.05 (0.73)	0.14 (0.37)	0.07 (0.64)	0.05 (0.74)	0.17 (0.30)

ABSI = A body shape index, AVI = Abdominal volume index, BAI = Body adiposity index, BFM = Body fat mass, BMC = Bone mineral content, BMI = Body mass index, BRI = Body roundness index, CI = Conicity index, FFM = Fat free mass, HC = Hip circumference, PBF = Percent body fat, PMM = Percent muscle mass, WC = Waist circumference, WHR = Waist to hip ratio, WHHR = Waist to hip to height ratio, WHtR = Waist to height ratio, WWI = Weight-adjusted-waist-index

^a^ ‘r’: Pearson correlation coefficient

^b^ P: significant value

Effect size = 0.86, confidence interval = 0.95

*. Correlation is significant at the 0.05 level (2-tailed)

With regards to previous studies, Age, gender, and physical activity were foremost covariates and differences were observed in our data. Therefore, our final model was adjusted for age, gender, and physical activity. Results of multiple linear regression revealed that there is a significant association between DIL and BMI (P = 0.04) in < 18 male soccer players, CI (P = 0.04) and WWI (P = 0.03) in ≥ 18 male soccer players, PBF (P = 0.02), PMM (P = 0.01) and WWI (P = 0.01) in ≥ 18 female soccer players. Nevertheless, no significant associations between DIL and body composition parameters were found in the referees. Additionally, this study showed a significant association between DII and BMC (P = 0.02) in male soccer referees, however, no significant associations were found in young soccer players and female athletes. More comprehensive information about the associations between DIL/DII and body composition parameters is presented in Table 4A and Table 4B, respectively. According to the significant relationship between some body composition parameters and dietary insulin load, it can be claimed that controlling dietary carbohydrates can be considered as a strategy to improve body composition in soccer players and referees.

**Table 4A:** The association between Dietary Insulin Load (DIL) and body composition adjusted for potential covariates

Categories of Athletes	Body composition parameters	Dietary Insulin Load		
		Unstandardized β coefficient	SE	P-value
**< 18 male soccer players** **(N = 12)**	**BMI (kg/m** ^**2**^ **)**	-0.00	0.00	0.04*
	**PBF (%)**	-0.00	0.00	0.45
	**PMM (%)**	0.00	0.00	0.45
	**WC (cm)**	0.00	0.00	0.45
	**HC (cm)**	-0.00	0.00	0.06
	**WHR**	-0.00	0.00	0.78
	**WHtR**	-0.00	0.00	0.27
	**ABSI**	0.00	0.00	0.68
	**AVI**	-0.00	0.00	0.15
	**BAI**	-0.00	0.00	0.41
	**BRI**	-0.00	0.00	0.26
	**CI**	-0.00	0.00	0.94
	**WWI**	0.00	0.00	0.83
	**WHHR**	0.00	0.00	0.80
	**BMC(kg)**	-0.00	0.00	0.07
**≥ 18 male soccer players** **(N = 11)**	**BMI (kg/m** ^**2**^ **)**	-0.00	0.00	0.99
	**PBF (%)**	0.00	0.00	0.11
	**PMM (%)**	-0.00	0.00	0.11
	**WC (cm)**	-0.00	0.00	0.11
	**HC (cm)**	-0.00	0.00	0.90
	**WHR**	0.00	0.00	0.06
	**WHtR**	0.00	0.00	0.19
	**ABSI**	0.00	0.00	0.07
	**AVI**	0.00	0.00	0.37
	**BAI**	0.00	0.00	0.89
	**BRI**	0.00	0.00	0.17
	**CI**	0.00	0.00	0.04*
	**WWI**	0.00	0.00	0.03*
	**WHHR**	0.00	0.00	0.09
	**BMC(kg)**	-0.00	0.00	0.62
**< 18 female soccer players** **(N = 24)**	**BMI (kg/m** ^**2**^ **)**	-0.00	0.00	0.59
	**PBF (%)**	0.00	0.00	0.89
	**PMM (%)**	-0.00	0.00	0.90
	**WC (cm)**	-0.00	0.00	0.86
	**HC (cm)**	-0.00	0.00	0.70
	**WHR**	0.00	0.00	0.93
	**WHtR**	-0.00	0.00	0.69
	**ABSI**	0.00	0.00	0.74
	**AVI**	-0.00	0.00	0.90
	**BAI**	-0.00	0.00	0.47
	**BRI**	-0.00	0.00	0.72
	**CI**	0.00	0.00	0.99
	**WWI**	-0.00	0.00	0.87
	**WHHR**	-0.00	0.00	0.90
	**BMC(kg)**	0.00	0.00	0.92
**≥ 18 female soccer players** **(N = 23)**	**BMI (kg/m** ^**2**^ **)**	-0.00	0.00	0.26
	**PBF (%)**	-0.00	0.00	0.02*
	**PMM (%)**	0.00	0.00	0.01*
	**WC (cm)**	-0.00	0.00	0.25
	**HC (cm)**	-0.00	0.00	0.83
	**WHR**	-0.00	0.00	0.10
	**WHtR**	-0.00	0.00	0.21
	**ABSI**	-0.00	0.00	0.61
	**AVI**	-0.00	0.00	0.27
	**BAI**	0.00	0.00	0.59
	**BRI**	-0.00	0.00	0.21
	**CI**	-0.00	0.00	0.15
	**WWI**	-0.00	0.00	0.01*
	**WHHR**	-0.00	0.00	0.13
	**BMC(kg)**	0.00	0.00	0.06
**Male soccer referees** **(N = 90)**	**BMI (kg/m** ^**2**^ **)**	0.00	0.00	0.67
	**PBF (%)**	-0.00	0.00	0.39
	**PMM (%)**	0.00	0.00	0.39
	**WC (cm)**	-0.00	0.00	0.26
	**HC (cm)**	-0.00	0.00	0.87
	**WHR**	-0.00	0.00	0.13
	**WHtR**	-0.00	0.00	0.68
	**ABSI**	-0.00	0.00	0.06
	**AVI**	-0.00	0.00	0.28
	**BAI**	0.00	0.00	0.27
	**BRI**	-0.00	0.00	0.68
	**CI**	-0.00	0.00	0.10
	**WWI**	-0.00	0.00	0.33
	**WHHR**	-0.00	0.00	0.72
	**BMC(kg)**	-0.00	0.00	0.59
**Female soccer referees** **(N = 39)**	**BMI (kg/m** ^**2**^ **)**	-0.00	0.00	0.97
	**PBF (%)**	0.00	0.00	0.82
	**PMM (%)**	-0.00	0.00	0.82
	**WC (cm)**	0.00	0.00	0.35
	**HC (cm)**	0.00	0.00	0.99
	**WHR**	0.00	0.00	0.11
	**WHtR**	0.00	0.00	0.46
	**ABSI**	0.00	0.00	0.09
	**AVI**	0.00	0.00	0.39
	**BAI**	-0.00	0.00	0.89
	**BRI**	0.00	0.00	0.49
	**CI**	0.00	0.00	0.16
	**WWI**	0.00	0.00	0.25
	**WHHR**	0.00	0.00	0.30
	**BMC(kg)**	-0.00	0.00	0.91

ABSI = A body shape index, AVI = Abdominal volume index, BAI = Body adiposity index, BFM = Body fat mass, BMC = Bone mineral content, BMI = Body mass index, BRI = Body roundness index, CI = Conicity index, FFM = Fat free mass, HC = Hip circumference, PBF = Percent body fat, PMM = Percent muscle mass, WC = Waist circumference, WHR = Waist to hip ratio, WHHR = Waist to hip to height ratio, WHtR = Waist to height ratio, WWI = Weight-adjusted-waist-index

A multiple linear regression model was created with adjustment for age, gender and physical activity level

P-value is considered significant at < 0.05

Effect size = 0.86, confidence interval = 0.95

**Table 4B:** The association between Dietary Insulin Index (DII) and body composition adjusted for potential covariates

Categories of Athletes	Body composition parameters	Dietary Insulin Index		
		Unstandardized β coefficient	SE	P-value
**< 18 male soccer players** **(N = 12)**	**BMI (kg/m** ^**2**^ **)**	0.05	0.09	0.57
	**PBF (%)**	-0.06	0.25	0.80
	**PMM (%)**	0.06	0.25	0.80
	**WC (cm)**	-0.09	0.28	0.75
	**HC (cm)**	0.05	0.23	0.82
	**WHR**	-0.00	0.00	0.48
	**WHtR**	0.00	0.00	0.89
	**ABSI**	0.00	0.00	0.49
	**AVI**	-0.02	0.08	0.80
	**BAI**	0.13	0.07	0.12
	**BRI**	0.00	0.02	0.88
	**CI**	-0.00	0.00	0.56
	**WWI**	-0.00	0.02	0.80
	**WHHR**	-0.00	0.00	0.95
	**BMC(kg)**	-0.00	0.02	0.86
**≥ 18 male soccer players** **(N = 11)**	**BMI (kg/m** ^**2**^ **)**	-0.04	0.17	0.80
	**PBF (%)**	-0.20	0.27	0.47
	**PMM (%)**	0.21	0.27	0.46
	**WC (cm)**	-0.11	0.42	0.79
	**HC (cm)**	0.12	0.33	0.73
	**WHR**	-0.00	0.00	0.43
	**WHtR**	-0.00	0.00	0.23
	**ABSI**	0.00	0.00	0.59
	**AVI**	-0.03	0.12	0.79
	**BAI**	-0.19	0.14	0.23
	**BRI**	-0.04	0.02	0.22
	**CI**	-0.00	0.00	0.55
	**WWI**	-0.03	0.02	0.20
	**WHHR**	-0.00	0.00	0.08
	**BMC(kg)**	0.03	0.04	0.49
**< 18 female soccer players** **(N = 24)**	**BMI (kg/m** ^**2**^ **)**	0.03	0.06	0.63
	**PBF (%)**	0.17	0.16	0.31
	**PMM (%)**	-0.17	0.16	0.31
	**WC (cm)**	0.24	0.16	0.15
	**HC (cm)**	0.06	0.12	0.60
	**WHR**	0.00	0.00	0.19
	**WHtR**	0.00	0.00	0.39
	**ABSI**	0.00	0.00	0.26
	**AVI**	0.06	0.04	0.16
	**BAI**	-0.04	0.07	0.53
	**BRI**	0.01	0.01	0.40
	**CI**	0.00	0.00	0.16
	**WWI**	0.01	0.01	0.41
	**WHHR**	0.01	0.01	0.56
	**BMC(kg)**	0.00	0.01	0.58
**≥ 18 female soccer players** **(N = 23)**	**BMI (kg/m** ^**2**^ **)**	0.02	0.04	0.61
	**PBF (%)**	0.06	0.10	0.52
	**PMM (%)**	-0.06	0.10	0.52
	**WC (cm)**	-0.00	0.12	0.98
	**HC (cm)**	-0.02	0.07	0.72
	**WHR**	0.00	0.00	0.80
	**WHtR**	0.00	0.00	0.67
	**ABSI**	-0.00	0.00	0.42
	**AVI**	-0.00	0.03	0.94
	**BAI**	-0.06	0.09	0.51
	**BRI**	-0.00	0.01	0.63
	**CI**	0.00	0.00	0.35
	**WWI**	0.00	0.00	0.38
	**WHHR**	0.00	0.00	0.74
	**BMC(kg)**	-0.00	0.00	0.42
**Male soccer referees** **(N = 90)**	**BMI (kg/m** ^**2**^ **)**	0.02	0.02	0.18
	**PBF (%)**	-0.01	0.04	0.72
	**PMM (%)**	0.01	0.04	0.73
	**WC (cm)**	0.11	0.06	0.08
	**HC (cm)**	0.07	0.04	0.09
	**WHR**	0.00	0.00	0.29
	**WHtR**	0.00	0.00	0.62
	**ABSI**	0.00	0.00	0.96
	**AVI**	0.03	0.02	0.07
	**BAI**	-0.02	0.02	0.31
	**BRI**	0.00	0.00	0.62
	**CI**	0.00	0.00	0.36
	**WWI**	0.00	0.00	0.97
	**WHHR**	0.00	0.00	0.58
	**BMC(kg)**	0.01	0.00	0.02*
**Female soccer referees** **(N = 39)**	**BMI (kg/m** ^**2**^ **)**	-0.01	0.05	0.79
	**PBF (%)**	0.03	0.10	0.72
	**PMM (%)**	-0.03	0.10	0.72
	**WC (cm)**	0.09	0.10	0.37
	**HC (cm)**	0.00	0.08	0.96
	**WHR**	0.00	0.00	0.12
	**WHtR**	0.00	0.00	0.63
	**ABSI**	0.00	0.00	0.07
	**AVI**	0.02	0.03	0.38
	**BAI**	-0.03	0.06	0.58
	**BRI**	0.00	0.01	0.63
	**CI**	0.00	0.00	0.23
	**WWI**	0.00	0.00	0.42
	**WHHR**	0.00	0.00	0.48
	**BMC(kg)**	0.00	0.00	0.50

ABSI = A body shape index, AVI = Abdominal volume index, BAI = Body adiposity index, BFM = Body fat mass, BMC = Bone mineral content, BMI = Body mass index, BRI = Body roundness index, CI = Conicity index, FFM = Fat free mass, HC = Hip circumference, PBF = Percent body fat, PMM = Percent muscle mass, WC = Waist circumference, WHR = Waist to hip ratio, WHHR = Waist to hip to height ratio, WHtR = Waist to height ratio, WWI = Weight-adjusted-waist-index

A multiple linear regression model was created with adjustment for age, gender and physical activity level

P-value is considered significant at < 0.05

Effect size = 0.86, confidence interval = 0.95

## Discussion

Given our results, there is distinctly discovered a significant positive correlation between dietary insulin load (DIL) and different body composition parameters including body mass index (BMI) in < 18 male soccer players, conicity index (CI), weight-adjusted waist index (WWI) in ≥ 18 male soccer players, percent body fat (PBF) and negative correlation with percent muscle mass (PMM) in ≥ 18 female soccer players. Conversely, body composition parameters had no overall significant correlation with DIL in referees. This novel finding concerning soccer players and referees is unprecedented. Altogether, our novel finding declares Sports, specifically, playing soccer affect body compositions and consequently accounted for better DIL, lower PBF, and higher PMM. Regarding previous studies, the decrease in the level of fat mass is linked to the reduction of free fatty acid in circulation; this process limits the access of skeletal muscle tissue to free fatty acid [49]. In addition, studies have shown that higher plasma insulin levels are associated with a higher percentage of body fat [50].

Insulin secretion occurs in response to the food, which directly reflects the dietary insulin index. Also, in comparison to the dietary glycemic index and glycemic load, the dietary insulin index is more suitable to quantify the relationship of insulin exposure and non-communicable diseases. Furthermore, insulin secretion primarily takes place after carbohydrate intake and even the combination of protein and carbohydrate plays a role in insulin secretion. This combination synergically leads to a raising insulin concentration and decreasing glycemia. Although fat does not reduce insulin response, it does lower glycemia [2, 45]. Some in vitro studies have shown that higher levels of IGF-1 play a role in the proliferation of preadipocytes. This mechanism causes body fat formation. Also, lipogenesis betides with absorbing cellular glucose by IGF-1 stimulation in preadipocytes and adipocytes and inhibiting lipolysis in body fat mass. We hypothesize that insulin resistance and increased IGF-1 concentrations predispose to postprandial insulinemic spikes and are related to fat accumulation in adipocytes [33].

Consistent with our findings, prior studies reported no significant association between DII with overweight and obesity in men, but, this association was significant in women [51]. Furthermore, a study among Iranian adults indicated that DIL and DII had no relationship with the risk of metabolic syndrome [52]. Moreover, some studies have conclusively established that there is a significant correlation between CI and fasting insulin levels among healthy premenopausal women [53]. In addition, Maysa et al. showed a significant correlation between insulin actions and insulin sensitivity in soccer players with type 2 diabetes [28]. Unlike our finding, Nassis et al. attempted to perform a study on obese and overweight with aerobic training, and they did not find any association between 12 weeks of aerobics training with body fat and body weight. However, they found that aerobic training ameliorates metabolic abnormalities in children [54]. Meng-Meng Liu et al. in accord with our results corroborated a significant correlation between insulin release at each phase and WHR [55].

The strength of our study is the novelty and lack of prior exploration of the association between DII and DIL with body compositions among soccer players and referees in both sexes. In addition, the determined sample size was considered very large in order to prevent over- or under-reporting by some individuals and rule out the possibility of missing some information.

Well-qualified analyses were controlled for various probable confounders to accomplish an independent association between DII and DIL with body compositions. Nevertheless, several limitations have to be deemed, first, based on its cross-sectional nature, causal inference is precluded and relies on a specific time period, which can contain assorted misinterpretations. Prospective studies are required to clarify their cause-and-effect relationship. Secondly, the existence of some unknown confounding factors should not be disaffirmed; they can erroneously affect the results. Also, the more reliable instrument for measuring body compositions is Dual-Energy X-Ray Absorptiometry (DEXA) and ‘Skinfolds’ methods than BIA. Furthermore, it is recommended that future studies consider this item for improved reliability in measuring body compositions [56, 57].

## Conclusion

This study demonstrates that DIL is positively associated with BMI, CI, and WWI in male soccer players and PBF, and WWI in female soccer players. Although, there was an observed negative association between DIL and PMM in males. In addition, a significant negative association between DII and BMC was observed in male soccer players.

## Data Availability

“This manuscript data and materials provide by requesting reliable email to corresponding authors”.
